# Lifestyle, genetic risk and incidence of cancer: a prospective cohort study of 13 cancer types

**DOI:** 10.1093/ije/dyac238

**Published:** 2023-01-18

**Authors:** Stephanie Byrne, Terry Boyle, Muktar Ahmed, Sang Hong Lee, Beben Benyamin, Elina Hyppönen

**Affiliations:** Australian Centre for Precision Health, University of South Australia, Adelaide, SA, Australia; UniSA Allied Health & Human Performance, University of South Australia, Adelaide, SA, Australia; South Australian Health and Medical Research Institute, Adelaide, SA, Australia; Australian Centre for Precision Health, University of South Australia, Adelaide, SA, Australia; UniSA Allied Health & Human Performance, University of South Australia, Adelaide, SA, Australia; South Australian Health and Medical Research Institute, Adelaide, SA, Australia; Australian Centre for Precision Health, University of South Australia, Adelaide, SA, Australia; South Australian Health and Medical Research Institute, Adelaide, SA, Australia; UniSA Clinical and Health Sciences, University of South Australia, Adelaide, SA, Australia; Department of Epidemiology, Faculty of Public Health, Jimma University Institute of Health, Jimma, Ethiopia; Australian Centre for Precision Health, University of South Australia, Adelaide, SA, Australia; UniSA Allied Health & Human Performance, University of South Australia, Adelaide, SA, Australia; South Australian Health and Medical Research Institute, Adelaide, SA, Australia; Australian Centre for Precision Health, University of South Australia, Adelaide, SA, Australia; UniSA Allied Health & Human Performance, University of South Australia, Adelaide, SA, Australia; South Australian Health and Medical Research Institute, Adelaide, SA, Australia; Australian Centre for Precision Health, University of South Australia, Adelaide, SA, Australia; South Australian Health and Medical Research Institute, Adelaide, SA, Australia; UniSA Clinical and Health Sciences, University of South Australia, Adelaide, SA, Australia

**Keywords:** Cancer, lifestyle, genetic risk, diet, obesity, physical activity, smoking, alcohol consumption

## Abstract

**Background:**

Genetic and lifestyle factors are associated with cancer risk. We investigated the benefits of adhering to lifestyle advice by the World Cancer Research Fund (WCRF) with the risk of 13 types of cancer and whether these associations differ according to genetic risk using data from the UK Biobank.

**Methods:**

In 2006–2010, participants aged 37–73 years had their lifestyle assessed and were followed up for incident cancers until 2015–2019. Analyses were restricted to those of White European ancestry with no prior history of malignant cancer (*n* = 195 822). Polygenic risk scores (PRSs) were computed for 13 cancer types and these cancers combined (‘overall cancer’), and a lifestyle index was calculated from WCRF recommendations. Associations with cancer incidence were estimated using Cox regression, adjusting for relevant confounders. Additive and multiplicative interactions between lifestyle index and PRSs were assessed.

**Results:**

There were 15 240 incident cancers during the 1 926 987 person-years of follow-up (median follow-up = 10.2 years). After adjusting for confounders, the lifestyle index was associated with a lower risk of overall cancer [hazard ratio per standard deviation increase (95% CI) = 0.89 (0.87, 0.90)] and of eight specific cancer types. There was no evidence of interactions on the multiplicative scale. There was evidence of additive interactions in risks for colorectal, breast, pancreatic, lung and bladder cancers, such that the recommended lifestyle was associated with greater change in absolute risk for persons at higher genetic risk (*P *<* *0.0003 for all).

**Conclusions:**

The recommended lifestyle has beneficial associations with most cancers. In terms of absolute risk, the protective association is greater for higher genetic risk groups for some cancers. These findings have important implications for persons most genetically predisposed to those cancers and for targeted strategies for cancer prevention.

Key Messages

- Common genetic variations captured by polygenic risk scores (PRSs) are associated with the risk of multiple cancers, providing opportunities for the identification of high-risk groups.
- We observed positive additive interactions in the associations between an index of recommended lifestyle and PRSs, consistent with some lifestyles being more beneficially associated with the risk of colorectal, breast, pancreatic, lung and bladder cancers in those at higher genetic risk.
- If the recommended lifestyle factors are causally related to risk, our data suggest that screening for genetic predisposition followed by lifestyle intervention might provide a viable strategy for targeted risk mitigation for some cancers.

## Introduction

Cancer is the second leading cause of death worldwide.^1^ Genetic and lifestyle factors play an important role in the aetiology of cancer. While the heritability of cancer overall has been estimated to be 33%,^2^ individual genetic variants typically have little impact. However, when assessed collectively using a polygenic risk score (PRS), a greater number of these genetic variants can substantially increase the likelihood of developing some cancers. Indeed, a high genetic risk (top 20%) has been reported to account for up to 30% of cases, although this varies by cancer type.^3^^,^^4^

An estimated 30–50% of all cancer cases could be prevented through lifestyle changes, such as eating more fruit, vegetables and wholegrains, and less red and processed meat; being physically active; maintaining a normal bodyweight; and avoiding tobacco and alcohol.^1^ These lifestyle factors often coexist, creating issues in estimating independent relationships with disease outcomes. Thus, research has assessed lifestyle factors collectively in a lifestyle score.^5^ While there is strong evidence that a lifestyle that adheres to these recommendations reduces the risk of colorectal and breast cancer, the evidence for other cancer types is less clear.^6–8^

Recent evidence suggests that lifestyle may help reduce cancer risk, even among those with a higher genetic risk of cancer.^9^ The strongest evidence has been obtained from studies on breast and colorectal cancer, which suggest lifestyle associations persist regardless of genetic risk,^10–12^ and adherence to lifestyle recommendations for cancer prevention may even be of greater benefit for those at higher genetic risk.^13^^,^^14^ However, these associations have not been explored for other cancer types. In this study, we investigate whether adhering to lifestyle cancer prevention recommendations by the World Cancer Research Fund (WCRF) is associated with the risk of 13 different types of cancer: bladder, breast, colorectal, kidney, lymphocytic leukaemia, lung, melanoma, non-Hodgkin’s lymphoma (NHL), oral cavity/pharyngeal, ovarian, pancreatic, prostate and uterine cancer. Our aim was to further determine whether the relationships between lifestyle and cancer differ by genetic risk (multiplicative interaction, i.e. whether the combined effect of lifestyle and genetic risk is different to the product of their individual effects on the relative risk scale), and whether a particular population group may benefit more from lifestyle intervention programmes (additive interaction, i.e. the combined effect of lifestyle and genetic risk on the risk of cancer is different to the sum of their individual effects on the absolute risk scale).^15^ This knowledge will inform cancer-prevention intervention strategies.

## Methods

### Study design and study population

This prospective cohort study utilizes data from the UK Biobank, which is a large population-based cohort of >500 000 adults aged 37–73 years and living in the UK at the time of the initial assessment in 2006–2010.^16^ Participants attended 1 of 22 assessment centres across England, Scotland and Wales, and completed an assessment including a questionnaire, anthropometric measures and collection of biological samples. UK Biobank was approved by the National Health Service North West Multi-centre Research Ethics Committee (11/NW/0382), the National Information Governance Board for Health and Social Care in England and Wales, and the Community Health Index Advisory Group in Scotland. All participants provided written informed consent.

**Figure 1:**
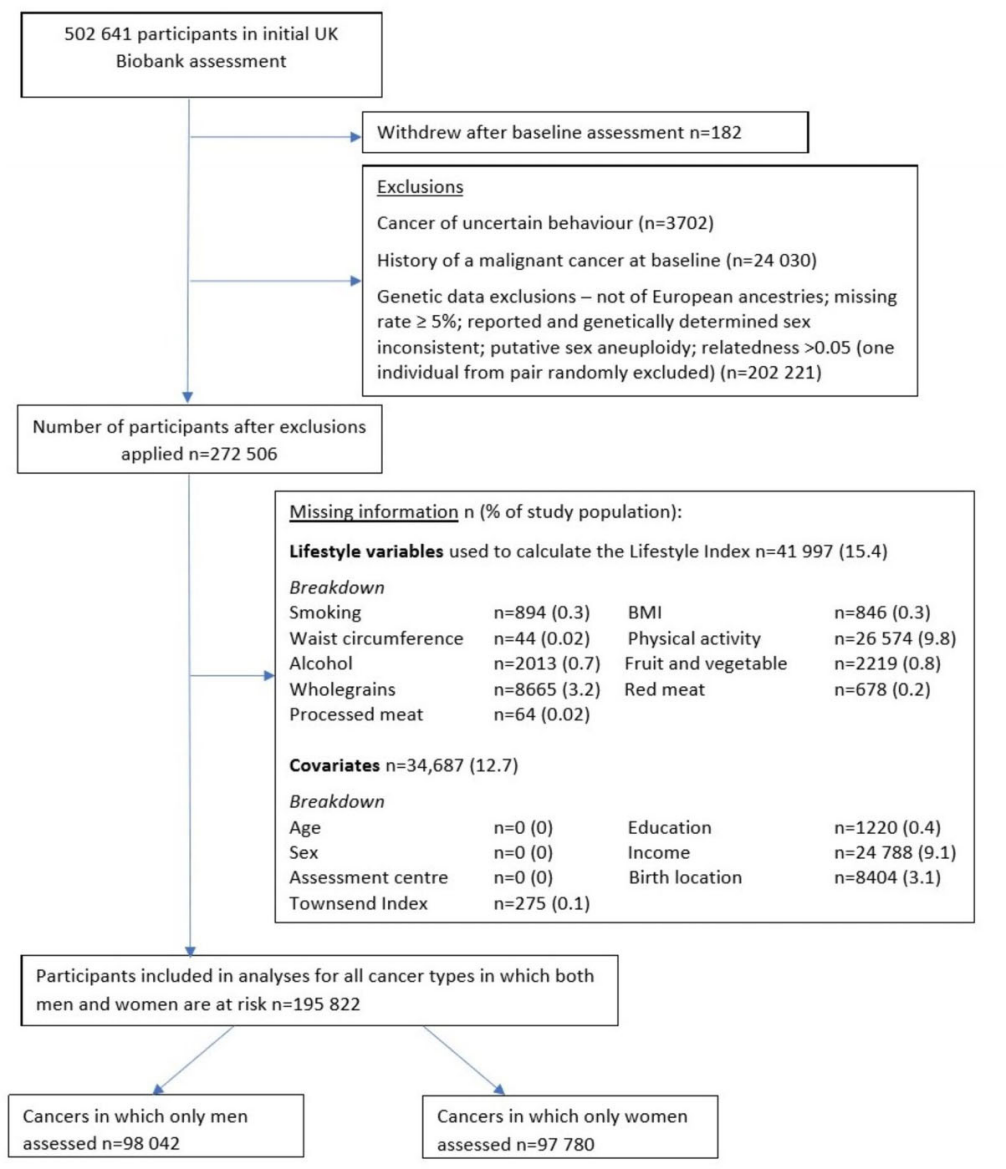
Ascertainment of the study sample included in the analyses

To reduce the effects of population stratification, our analyses were restricted to participants of White European ancestries and we included participants with no history of any malignant cancer at baseline and sufficient data available for the lifestyle factors and other covariates of interest (Figure 1).

### PRSs

We calculated PRSs for each participant for 13 cancer types based on single-nucleotide polymorphisms (SNPs) selected by Graff *et al.*^3^ Briefly, they identified a set of independent SNPs associated with cancer risk for each cancer type from previously published cancer genome-wide association studies. SNP selection was restricted to those that are common and available in the UK Biobank data. SNPs with the strongest associations with the broadest phenotype were preferentially selected, with an overall linkage disequilibrium *r*^2^ < 0.3 to ensure independence (see Supplementary Table S1, available as Supplementary data at *IJE* online, for the number of SNPs used in the PRS calculations; see Graff *et al.* for a full list of genetic variants^3^).

We calculated PRSs as the sum of the number of risk alleles multiplied by the log of the odds ratio (OR) for each SNP, as implemented in PLINK software^17^ using the following formula: where *n* is the number of SNPs in the set of independent SNPs for that cancer type, *x_il_* is the number of risk alleles (0, 1 or 2) for the *i*-th individual at the *l*-th SNP and *OR_l_* is the estimated OR for the *l*-th SNP using a logistic regression.


$${\mathrm{PRS}}_{i}=\sum_{l=1}^{n}x_{il}l\mathrm{og}({\mathrm{OR}}_{l})$$

Each PRS was z-standardized, with higher scores representing a greater genetic risk. A PRS for overall cancer was also derived as the sum of the z-standardized PRSs for the 13 cancer types, each weighted according to the distribution of incident cases of these cancers reported by Cancer Research UK in 2017 (https://www.cancerresearchuk.org/health-professional/cancer-statistics/incidence#heading-Four).

### Lifestyle index

A lifestyle index was constructed based on the WCRF/American Institute for Cancer Research (WCRF/AICR) cancer prevention recommendations and standardized scoring system (full details of the calculation of the lifestyle index are in Supplementary Table S2, available as Supplementary data at *IJE* online).^5^ Baseline anthropometric and touchscreen questionnaire information was available for five of the eight recommendations included in the 2018 WCRF/AICR score (normal weight; physical activity; wholegrain, fruit and vegetable intake; red and processed meat intake; alcohol consumption). Smoking status (categorized as never, former or current smoking) was also included in the lifestyle index, given its relevance as a modifiable lifestyle risk factor for multiple cancers. The lifestyle index ranged from 0 to 6, with higher scores indicating greater adherence to the recommendations.

### Cancer outcome ascertainment

Malignant cancer diagnoses for each cancer type were identified using the 9th and 10th revisions of the International Classification of Diseases (ICD-9 and ICD-10) codes, phenotype and tumour behaviour information obtained through linkage to national cancer registries in England, Wales and Scotland. Incident cancer events were those diagnosed after baseline assessment. Cancer information was complete up to 31 July 2019 for England and Wales, and 31 October 2015 for Scotland. Hence, follow-up time was defined as being from the date of baseline assessment to the earliest of the date of diagnosis, date of death (obtained via linkage to death registries) or 31 July 2019 for England and Wales residents and 31 October 2015 for Scotland residents.

### Statistical analysis

Baseline characteristics were summarized for the total study population, those diagnosed with any of the 13 cancers examined (referred to as ‘overall cancer’) and individually for each cancer type. Characteristics were summarized as percentages for all variables for greater interpretability.

Cox proportional hazard regression models were used to investigate associations between PRS, lifestyle index and incidence of overall cancer, and separately for each cancer. PRSs and lifestyle index were used as continuous variables, standardized to estimate hazard ratio per SD change. Incidences of cancers that are sex-specific were assessed in the relevant sex only (female cancers—ovarian, uterine, breast; male cancers—prostate). Breast cancer analyses were further restricted to post-menopausal women only (self-reported at baseline). All models were adjusted for age at baseline (continuous); sex (where relevant); assessment centre; socio-economic status (Townsend Index—continuous); education [none listed, General Certificate of Secondary Education (GCSE)/Certificate of Secondary Education (CSE) or equivalent, A-levels or equivalent, college/university or other professional training]; household income (<18 000, 18 000–30 999, 31 000–51 999, 52 000–100 000, >100 000); birth location (north south coordinate and east west coordinate—both in deciles) and population stratification measured by the first 40 principal components. Models with and without mutual adjustment for lifestyle index/PRS were examined. Subsequent analyses adjusting for additional covariates specific to each cancer type were also performed (see Supplementary Table S3, available as Supplementary data at *IJE* online, for a list of the additional covariates and definitions). Additional analyses exploring the risk of cancer in the top 5% of PRSs were conducted using Cox regression with the bottom tertile of PRSs as the reference and adjusting for the same covariates.

For each cancer outcome, we investigated multiplicative interactions between lifestyle and genetic risk by using a likelihood ratio test to compare Cox models with and without an interaction term between PRS and lifestyle index. Meanwhile, additive interactions were assessed by using the relative excess risk due to interaction (RERI), which was calculated from Cox models with the interaction term.^15^ For these models, the lifestyle index and PRSs were categorized into tertiles for greater interpretability [PRS—lower (lowest tertile), intermediate (middle tertile) and higher (highest tertile) genetic risk; lifestyle index—lower (range 0–3), intermediate (range 3.25–3.75) and higher (range 4–6) lifestyle index, representing lower, intermediate and higher adherence to lifestyle recommendations]. Observed interactions were further explored visually by using a forest plot produced from a multivariable Cox proportional hazards model with a combined genetic and lifestyle risk variable (nine categories with lower genetic risk and higher lifestyle index as the reference). As smoking is a strong risk factor for lung cancer, we performed sensitivity analyses removing smoking from the lifestyle index and including it as a confounder for this outcome. Proportionality of hazards assumptions were verified by using Schoenfeld residuals. Two-sided *P*-values of ≤0.05 were considered as evidence of an association. A *P*-value threshold of 0.0036 (calculated as 0.05/number of cancer outcomes) was applied to interaction models to account for multiple testing. All analyses were performed using Stata SE version 16 (StataCorp, College Station, TX). Results are presented in order of power determined from the number of cases for each cancer type.

## Results

A summary of the baseline characteristics of the 195 822 participants included in the analysis and for those diagnosed with any of the 13 types of cancer during the follow-up period are provided in Table 1. Over the 1 926 987 person-years of follow-up [median (interquartile range) length of follow-up = 10.2 (9.4–10.9) years], there was a total of 15 240 incident cases of the 13 cancer types of interest. The cancers with the highest number of incident cases were prostate cancer, colorectal cancer and post-menopausal breast cancer. Generally, risk of cancer was higher in men, those of older age and those who have lower levels of education and income. Lower adherence to lifestyle recommendations and a higher genetic predisposition were also associated with a higher incidence of most, but not all, cancers.

**Table 1:** Baseline characteristics of the total study population and of incident cases of each cancer type

Characteristic	Total study population	Overall cancer	Prostate cancer	Colorectal cancer	Post-menopausal breast cancer	Lung cancer	Melanoma	Non-Hodgkin’s lymphoma	Kidney cancer	Uterine cancer	Pancreatic cancer	Bladder cancer	Oral cavity/pharyngeal cancer	Ovarian cancer	Lymphocytic leukaemia
*N*	195 822	15 240	4476	2150	1990	1256	1200	721	547	494	451	424	415	315	246
Age at baseline assessment (%)^a^															
≤50 years	27.0	11.5	4.8	9.8	2.1	5.3	17.5	9.2	8.4	11.1	6.7	3.3	13.7	20.0	5.7
51–60 years	36.7	33.1	29.4	32.5	45.5	30.3	33.5	32.3	33.8	41.9	29.7	23.8	40.5	30.8	31.7
≥61 years	36.4	55.4	65.8	57.7	52.4	64.4	49.0	58.5	57.8	47.0	63.6	72.9	45.8	49.2	62.6
Female (%)	49.9	43.5	0	38.8	100	44.2	45.7	41.3	33.8	100	41.0	17.5	29.2	100	29.7
Townsend Index (%)^a^															
Quintile 1	22.5	22.6	24.6	22.8	22.5	15.2	26.0	25.4	24.3	17.0	22.4	21.0	16.4	20.6	24.0
Quintile 2	21.6	22.3	23.2	23.7	21.8	19.2	25.0	19.7	21.0	23.9	23.1	24.5	22.7	24.8	17.5
Quintile 3	20.7	20.6	21.6	19.7	20.5	17.8	19.9	21.8	24.5	19.6	22.6	19.6	17.4	19.1	26.8
Quintile 4	19.4	18.9	18.1	18.0	20.9	20.1	17.5	18.9	15.7	21.1	15.5	18.2	21.5	20.6	17.5
Quintile 5	15.8	15.6	12.6	15.8	14.4	27.7	11.6	14.3	14.4	18.4	16.4	16.8	22.2	14.9	14.2
Education (%)^a^															
None listed	12.6	16.3	15.5	15.7	15.1	30.0	13.2	18.5	18.1	13.4	19.3	21.0	19.0	15.6	20.7
GCSE/CSE or equivalent	27.1	25.5	21.6	26.3	29.2	25.1	27.7	22.2	28.0	31.4	22.4	27.1	26.3	31.4	24.8
A-levels or equivalent	12.3	11.3	10.5	11.8	12.2	10.0	10.9	10.8	11.9	10.5	9.5	8.5	12.1	11.4	8.9
College/university or other professional training	48.0	47.0	52.4	46.2	43.6	35.0	48.3	48.5	42.1	44.7	48.8	43.4	42.7	41.6	45.5
Average total household income before tax (%)^a^															
<£18 000	19.4	23.8	20.4	23.8	25.7	41.2	18.3	22.6	24.7	25.3	27.7	29.0	28.9	27.9	30.1
£18 000 –30 999	25.2	28.4	27.7	29.7	31.7	30.1	23.8	32.6	29.8	32.0	27.3	30.7	25.8	31.1	26.4
£31 000–51 999	27.2	25.6	26.8	25.6	24.3	18.4	27.8	26.9	25.2	24.3	25.3	22.6	25.1	24.4	26.0
£52 000–100 000	22.4	17.4	19.6	16.0	15.2	8.1	24.3	13.5	16.3	15.2	14.6	13.9	16.6	12.4	15.0
>£100 000	5.9	4.8	5.5	4.9	3.1	2.2	5.8	4.4	4.0	3.2	5.1	3.8	3.6	4.1	2.4
Lifestyle index (%)^a^															
Lower	36.6	42.0	41.0	44.9	33.6	63.1	34.8	38.0	46.6	38.3	48.3	55.9	50.6	30.5	38.2
Intermediate	32.4	32.0	32.2	30.6	36.9	23.5	35.5	31.9	35.1	34.0	32.2	25.9	28.9	35.9	33.3
Higher	31.0	26.0	26.8	24.5	29.6	13.4	29.8	30.1	18.3	27.7	19.5	18.2	20.5	33.7	28.5
PRS (%)^a^															
Lower genetic risk	–	30.5	24.3	22.4	24.9	25.5	23.0	27.7	27.8	22.3	20.6	24.3	32.8	26.7	22.0
Intermediate genetic risk	–	33.3	31.2	32.9	33.1	32.9	31.2	33.3	32.2	36.2	33.3	36.6	33.0	34.0	24.8
Higher genetic risk	–	36.3	44.5	44.7	42.0	41.6	45.8	39.0	40.0	41.5	46.1	39.2	34.2	39.4	53.3

Overall cancer = overall incident cases of the 13 cancer types assessed in this study.

GCSE, General Certificate of Secondary Education; CSE, Certificate of Secondary Education; PRS, polygenic risk score.

a Percentages may not add up to 100% due to rounding.

**Figure 2:**
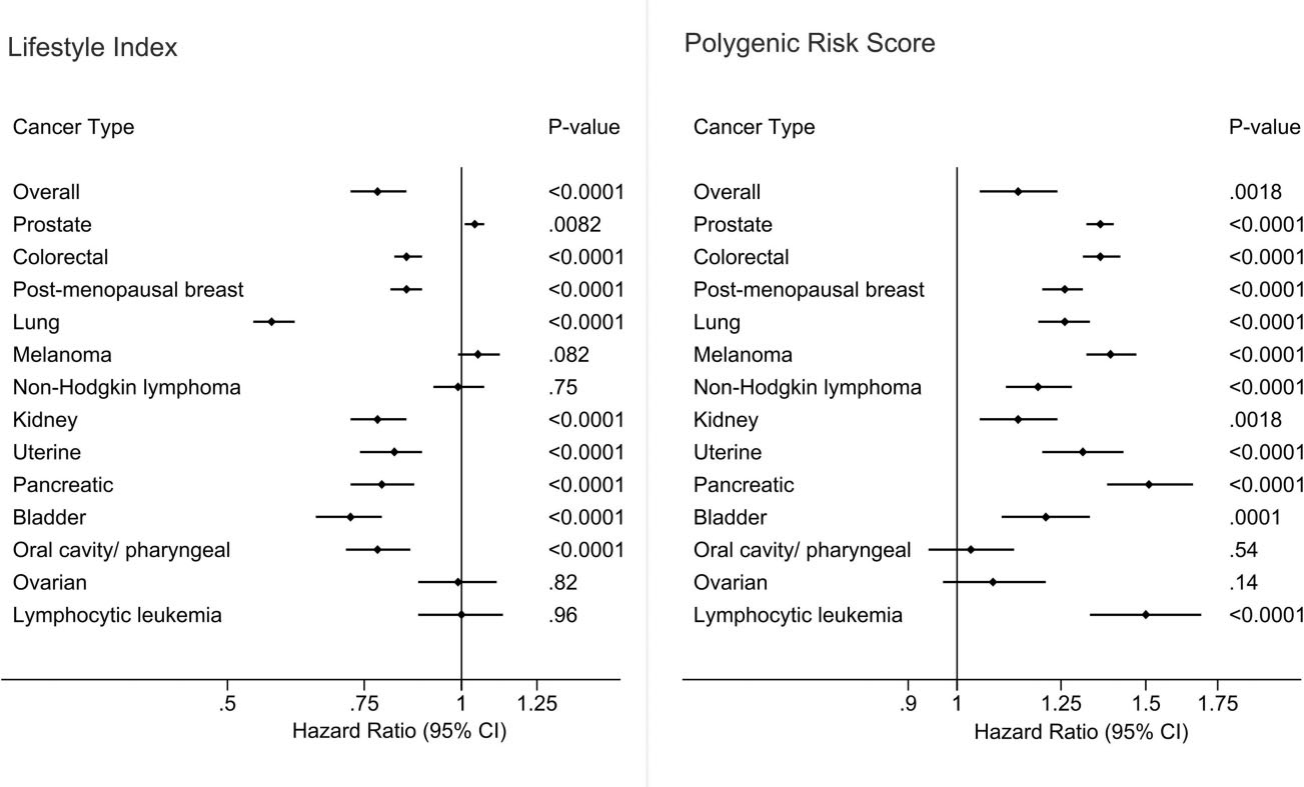
Adjusted associations between the lifestyle index, polygenic risk score and cancer. Cox proportional hazards regression adjusted for age at baseline, sex (where relevant), assessment centre, 40 principal components of ancestries, Townsend Index, education, birth location and income; overall cancer = overall incident cases of the 13 cancer types assessed in this study

After adjusting for confounders, a higher lifestyle index was associated with a lower risk of overall cancer [hazard ratio (HR) 95% CI per SD increase in lifestyle index = 0.89 (0.87, 0.90)], colorectal cancer [0.85 (0.82, 0.89)], post-menopausal breast cancer [0.85 (0.81, 0.89)], lung cancer [0.57 (0.54, 0.61)], kidney cancer [0.78 (0.72, 0.85)], uterine cancer [0.82 (0.74, 0.89)], pancreatic cancer [0.79 (0.72, 0.87)], bladder cancer [0.72 (0.65, 0.79)] and oral cavity/pharyngeal cancer [0.78 (0.71, 0.86)] (Figure 2). There was no association between the lifestyle index and melanoma, NHL, ovarian cancer and lymphocytic leukaemia. An association in the opposite direction was observed between the lifestyle index and prostate cancer risk [1.04 (1.01, 1.08)] (Figure 2). Estimates remained unchanged upon adjustment for PRS (Supplementary Table S4, available as Supplementary data at *IJE* online). Further adjustment for other potential confounders specific to cancer type had little effect on the estimates presented (Supplementary Table S4, available as Supplementary data at *IJE* online).

A higher PRS was associated with a higher risk of overall cancer and all cancer types assessed, except oral cavity/pharyngeal and ovarian cancers (Figure 2). The cancers with the highest risk with increasing PRS were prostate cancer [1.36 (1.32, 1.40)], colorectal cancer [1.36 (1.31, 1.42)], melanoma [1.39 (1.32, 1.47)], pancreatic cancer [1.51 (1.36, 1.66)] and lymphocytic leukaemia [1.50 (1.33, 1.69)]. Again, addition of the lifestyle index as a covariate had no effect on the estimates and further adjustment for other cancer-specific potential confounders also had little impact (Supplementary Table S4, available as Supplementary data at *IJE* online). Further exploration of genetic risk found that those in the top 5% of PRS for prostate, colorectal, melanoma and pancreatic cancers had an estimated 2.5-, 2.5-, 3- and 4-fold higher risk, respectively (Table 2).

**Table 2:** Risk of cancer for those in the top 5% of polygenic risk scores

Cancer type	Top 5% of polygenic risk scores		
	Number of cases	HR 95% CI^a^	*P*-value
Overall cancer	896	1.33 (1.24, 1.43)	<0.0001
Prostate cancer	383	2.53 (2.25, 2.84)	<0.0001
Colorectal cancer	180	2.52 (2.13, 3.00)	<0.0001
Post-menopausal breast cancer	225	1.92 (1.59, 2.32)	<0.0001
Lung cancer	104	2.22 (1.78, 2.77)	<0.0001
Melanoma	121	3.01 (2.43, 3.74)	<0.0001
Non-Hodgkin's lymphoma	61	2.06 (1.54, 2.74)	<0.0001
Kidney cancer	28	1.25 (0.83, 1.87)	0.28
Uterine cancer	35	2.22 (1.52, 3.25)	<0.0001
Pancreatic cancer	57	4.14 (2.97, 5.76)	<0.0001
Bladder cancer	31	1.99 (1.33, 2.98)	0.0008
Oral cavity/pharyngeal cancer	26	1.31 (0.86, 1.99)	0.21
Ovarian cancer	20	1.55 (0.95, 2.53)	0.078
Lymphocytic leukaemia	24	3.00 (1.85, 4.85)	<0.0001

a Reference category = bottom tertile of polygenic risk scores.

All models adjusted for lifestyle index category, age at baseline, sex, assessment centre, 40 principal components of ancestries, Townsend Index, education, birth location and income.

**Figure 3:**
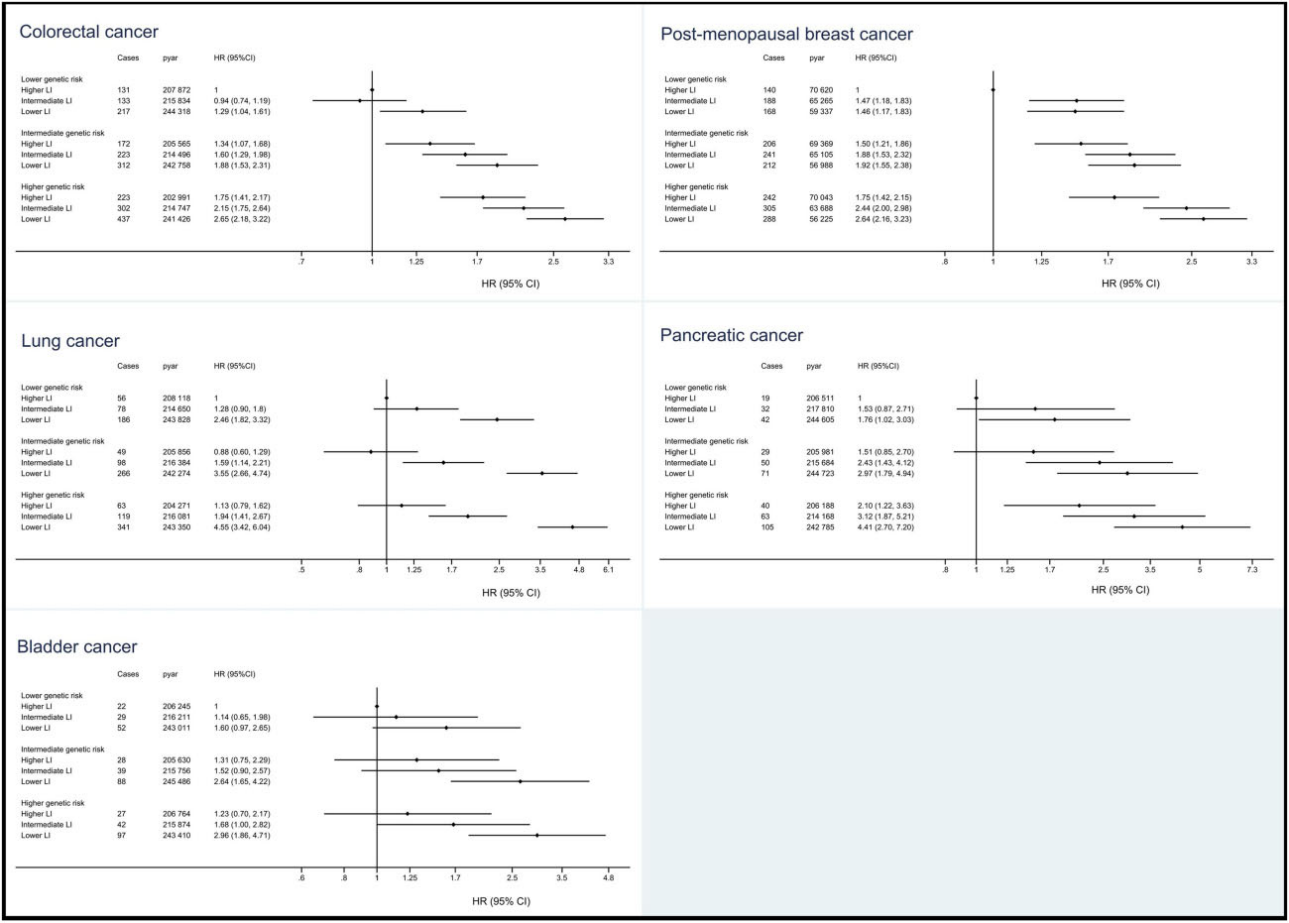
Risk of colorectal, post-menopausal breast, lung, pancreatic and bladder cancers according to genetic risk and lifestyle. Cox proportional hazards regression adjusted for age at baseline, sex (where relevant), assessment centre, 40 principal components of ancestries, Townsend Index, education, birth location and income; LI, lifestyle index; pyar, person-years at risk; HR, hazard ratio

There were no multiplicative interactions observed between lifestyle index and PRS for any of the cancer outcomes (*P *>* *0.03 for all outcomes after correction for multiple testing). However, some evidence for an additive interaction was observed for colorectal, breast, lung, pancreatic and bladder cancers (Supplementary Table S5, available as Supplementary data at *IJE* online). Forest plots of risk with a combined lifestyle/genetic risk variable were constructed to visually depict these interactions. These plots show a greater change in absolute risk across lifestyle tertiles in individuals with a higher genetic risk of these cancers (Figure 3). For lung and bladder cancers, there were no differences in cancer risk by PRS among persons with a higher lifestyle index.

We further investigated the association between lifestyle and lung cancer by removing smoking status from the lifestyle index and including it as a confounder. Although strongly attenuated, a higher lifestyle index was associated with a lower risk of lung cancer also when smoking was excluded [HR (95%CI) per SD increase = 0.91 (0.86, 0.97), *P *=* *0.0016; lifestyle index with smoking included HR = 0.57 (0.54, 0.61)]. The additive interaction between lifestyle index and PRS was attenuated by excluding information on smoking status [lower lifestyle index/higher genetic risk vs higher lifestyle index/lower genetic risk RERI = 0.44 (0.04, 0.85) *P *=* *0.033; *P*_trend_ = 0.021].

## Discussion

In this study, adhering to lifestyle advice by the WCRF was associated with a lower risk of several cancers and a higher genetic risk was associated with an increased risk. These associations were independent of each other. There were no multiplicative interactions between lifestyle and genetic risk on the relative risk scale, while we observed some evidence for risk differences (additive interaction) suggesting that adherence to lifestyle recommendations for cancer prevention may be of greater benefit in those with a higher genetic susceptibility to colorectal, breast and pancreatic cancers. We found that risks of lung and bladder cancers do not differ by genetic risk among individuals with higher adherence to lifestyle recommendations. Our findings are consistent with the limited comparable research for breast^10^^,^^13^ and colorectal cancers,^11^^,^^12^^,^^14^ and are novel for the other cancer types examined here. Communicating these results to groups with a higher genetic risk of cancer may help alleviate any distress experienced due to awareness of their increased risk and is likely to be empowering and potentially supportive of positive behavioural changes.

A higher genetic risk of cancer was associated with an increased risk of overall cancer and 11 cancer types: prostate, colorectal, breast (post-menopause), lung, melanoma, NHL, kidney, uterine, pancreatic, bladder and lymphocytic leukaemia. The highest genetic risk was observed for pancreatic cancer, with those in the top 5% of PRS having a 4-fold higher risk compared with those in the bottom tertile of genetic risk. This increase is similar in magnitude to *BRCA1* and *BRCA2* gene variants and breast cancer risk, which are far less common (prevalence of 0.2–0.3%) and trigger more frequent cancer screening.^18^ Pancreatic cancer is one of the leading causes of cancer death that is typically diagnosed at a late stage when the 5-year survival rate is <10%.^19^ Screening for pancreatic cancer is recommended for individuals deemed as high-risk^20^ and our results suggest a PRS could be used as an additional tool to assist in the identification of persons at high risk. We also found that persons with lower adherence to lifestyle recommendations had an increased risk of pancreatic cancer and there was a positive additive interaction on the risk difference scale, suggesting the combined association of lower adherence to lifestyle recommendations and higher genetic risk on the risk of pancreatic cancer is more than the sum of the individual associations.

Lower adherence to lifestyle recommendations was associated with a higher risk of overall cancer and eight cancer types: colorectal, post-menopausal breast, lung, kidney, uterine, pancreatic, bladder and oral cavity/pharyngeal cancers. These findings are consistent with previously published research for breast and colorectal cancers, and add to the limited and inconclusive research on overall lifestyle and other cancers.^6^^,^^7^ In addition to pancreatic cancer, we also observed additive interactions for colorectal, breast, lung and bladder cancers. If these lifestyle recommendations are causally related to risk of these cancers, our findings suggest that lifestyle changes in line with WCRF/AICR lifestyle recommendations may result in greater reductions in absolute risk of these cancers in high genetic risk populations compared with low genetic risk groups and, where resources are limited, lifestyle interventions should target these high genetic risk groups to maximize the potential reduction in cancer incidence. Further research is needed to establish a causal relationship; however, our findings support the current public health message that not smoking; avoiding alcohol; consuming a diet rich in wholegrains, fruit and vegetables, and low in red and processed meat; maintaining a normal bodyweight; and engaging in regular physical activity reduce cancer risk.

We found no association between the lifestyle recommendations and risk of melanoma, NHL, ovarian cancer and lymphocytic leukaemia. These results were not unexpected; none of the lifestyle factors included in the lifestyle index has been conclusively linked with the risk of lymphocytic leukaemia, melanoma or NHL and only obesity is positively associated with ovarian cancer risk.^8^ For melanoma, although evidence indicates that higher alcohol intake may increase risk, greater physical activity levels have also been associated with increased risk, which is likely related to higher sun exposure levels.^21^^,^^22^

We found that higher adherence to lifestyle recommendations was associated with a higher risk of prostate cancer. This finding is inconsistent with the majority of previous research suggesting either a negative association or no association for combined or individual components of the lifestyle index^6^^,^^23–27^ and may be the result of healthy volunteer bias.

This large prospective cohort study has some limitations. The length of the follow-up and age range of the study population have limited the number of incident cancer cases. Consequently, some statistical models may not have been adequately powered to observe a modifying effect of genetic risk on lifestyle–cancer associations. There are likely to be measurement errors in the components of the lifestyle score as they are almost all measured via self-report; however, this is likely to attenuate the findings to the null. We also cannot rule out the possibility of residual confounding, although we adjusted for multiple covariates. The measure of genetic risk was limited by the SNPs included in the PRS, which may not be exhaustive. Lastly, this study was conducted in adults of European ancestries, so the relevance of these findings to populations of other ethnicities is unclear.

In summary, our findings indicate that adhering to the WCRF/AICR lifestyle recommendations is associated with a lower cancer risk and, for some specific cancer types, greater adherence to lifestyle recommendations may be more beneficial among population groups with a higher genetic susceptibility to that cancer type. Our findings also highlight the potential benefit of using a PRS to identify those with a higher genetic risk of pancreatic cancer, and possibly other cancers, as PRSs are further refined. PRSs could be used in conjunction with other tools to increase the accuracy of identifying high-risk individuals who may then undergo regular screening and be targeted by prevention intervention strategies. This prospect requires further investigation using cohort studies conducted across various ethnic populations.

## Ethics approval

This study is a secondary analysis of UK Biobank data. The UK Biobank cohort study was approved by the National Health Service North West Multi-centre Research Ethics Committee (11/NW/0382), the National Information Governance Board for Health and Social Care in England and Wales, and the Community Health Index Advisory Group in Scotland. All participants provided written informed consent.

## Supplementary Material

dyac238_Supplementary_Data

## Data Availability

This research utilizes data from the UK Biobank resource (application number 20175), which is available directly from UK Biobank upon submission of a data request proposal. See https://www.ukbiobank.ac.uk.
