# Supplementary material for: Lifestyle, genetic risk and incidence of cancer: a prospective cohort study of 13 cancer types
Source: Int J Epidemiol. 2023 Jan 18;52(3):817–26. doi: 10.1093/ije/dyac238 (PMC10244040; doi:10.1093/ije/dyac238)
Supplement: dyac238_Supplementary_Data [file dyac238_supplementary_data.docx]

## **Supplementary material**

**Table S1 – Number of SNPs used to calculate the PRS for each cancer type**

| Cancer Type | Number of SNPs used in PRS calculation |
| --- | --- |
| Prostate | 161 |
| Colorectal | 103 |
| Breast | 187 |
| Lung | 109 |
| Melanoma | 24 |
| Non-Hodgkin lymphoma | 19 |
| Kidney | 19 |
| Uterine | 9 |
| Pancreatic | 22 |
| Bladder | 15 |
| Oral cavity/pharynx | 14 |
| Ovarian | 36 |
| Lymphocytic leukaemia | 75 |

SNP – single nucleotide polymorphism; PRS – polygenic risk score

**Table S2 - Definition of lifestyle factors**

| **Lifestyle factor** | **Measure** | **Classification** | Scoring |
| --- | --- | --- | --- |
| Be a healthy | BMI (kg/m2) | 18.5 to <25 | 0.5 |
| weight |  | 25 to <30 | 0.25 |
|  |  | <18.5 or ≥30 | 0 |
|  | Waist | Men: <94; Women: <80 | 0.5 |
|  | circumference | Men: 94 to <102; Women: 80 to <88 | 0.25 |
|  | (cm)^a^ | Men: ≥102; Women: ≥88 | 0 |
| Be physically | Physical activity | ≥3000 | 1 |
| active | (MET- | 600 to <3000 | 0.5 |
|  | mins/week)^b^ | <600 | 0 |
| Eat a diet rich in wholegrains, | Fruit and | ≥5 | 0.5 |
|  | vegetable intake | 3 to <5 | 0.25 |
| vegetables, fruit, and beans | (serves/day) | <3  (1 serve = 1 piece fresh fruit, 5 pieces dried fruit, or 3 heaped tablespoons of cooked or raw vegetables) | 0 |
|  | Wholegrain intake | ≥5.5 | 0.5 |
|  | (serves/day) | >2 to <5.5 | 0.25 |
|  |  | ≤2  (1 serve = 1 bowl cereal or 1 slice wholemeal or wholegrain bread. Cereal type not considered due to large amount of missingness. Wholegrain intake calculated without cereal type was slightly more correlated with dietary fibre intake from 24 hr diet recall than wholegrain intake calculated with cereal type considered) | 0 |
| Limit | Red meat intake | <2 times/week | 0.5 |
| consumption of | (times/week) | 2-4 times/week | 0.25 |
| red and |  | >4 times/week | 0 |
| processed meat | Processed meat | <once/week | 0.5 |
|  | intake | once/week | 0.25 |
|  | (times/week) | ≥twice/week | 0 |
| Limit alcohol | Alcohol intake | 0.0 | 1 |
| consumption | (g/day)^c^ | Males: >0 to ≤28; Females: >0 to ≤14 | 0.5 |
|  |  | Males: >28; Females: >14  (1 glass champagne/red wine/white wine = 12.8 g, 1 pint of beer = 18.4 g, 1 measure of spirits (25 ml) = 8 g, 1 standard measure of fortified wine (50 ml) = 8 g; 1 glass of other alcoholic drinks (e.g. alcopops) = 12 g.^1^ | 0 |
| Don’t smoke | Current Smoking | Never Smoker | 1 |
|  | Status | Former Smoker | 0.5 |
|  |  | Current Smoker | 0 |

^a^ Those with a waist circumference (WC) < 50 cm were excluded (i.e. WC recoded to missing; n=7)

^b^ Physical activity calculated as metabolic equivalent (MET)-mins/week using IPAQ short form guidelines^2^ and the self-reported estimated time spent (on a typical day) doing at least 2 of the 3 following activities – walking, moderate physical activity, and vigorous physical activity.

^c^ Estimated from self-reported frequency of alcohol intake and weekly or monthly quantities of different types of alcoholic beverages consumed. For those missing information on quantity of “other alcoholic drinks”, intake was estimated from the quantities reported for wine, beer, spirits, and fortified wine (n=224,609). For those who reported consuming alcohol less than weekly and had missing data on quantities of different beverage types, scores were estimated from data available (n=1,166) or, where there were no data on quantities consumed, a score of 0.5 was allocated (n=73,137).

**Table S3 – Definition of additional covariates included in further statistical models**

| **Cancer Type** | **Additional covariates included in Model 3** |
| --- | --- |
| Colorectal cancer | Height (cm), history of inflammatory bowel disease, Crohn’s disease, or ulcerative colitis (yes/no) |
| Breast cancer | Height (cm), ever hormone replacement therapy use (yes/no), ever oral contraceptive pill use (yes/no), number of live births, age at onset of menopause (last menstrual period; yes/no), age at menarche (years) |
| Uterine cancer | Height (cm), ever hormone replacement therapy use (yes/no), number of live births, have had menopause at baseline (yes/no), age at menarche (years) |
| Kidney cancer | Height (cm) |
| Lung cancer | History of chronic obstructive pulmonary disease or pulmonary fibrosis (yes/no) |
| Melanoma | Height (cm), time spent outdoors in summer (hours/day), use of sun/UV protection (never/rarely, sometimes, most of the time, always, do not go out in sunshine), solarium use (yes/no) |
| Non-Hodgkin lymphoma | History of any of the following autoimmune diseases – Sjogren syndrome, systemic lupus erythematosus, haemolytic anaemia, rheumatoid arthritis, pernicious anaemia, myasthenia gravis, celiac disease, immune thrombocytopenic purpura, inflammatory bowel disease, Crohn’s disease, ulcerative colitis, multiple sclerosis, systemic sclerosis or scleroderma, poly- or dermatomyositis, psoriasis, sarcoidosis, type 1 diabetes (yes/no) |
| Oral cavity/pharyngeal cancer | Time spent outdoors in summer (hours/day), use of sun/UV protection (never/rarely, sometimes, most of the time, always, do not go out in sunshine) |
| Ovarian cancer | Height (cm), ever hormone replacement therapy use (yes/no), ever oral contraceptive pill use (yes/no), number of live births, have had menopausal at baseline (yes/no), age at menarche (years), tubal ligation or fallopian tubes removed (yes/no) |
| Pancreatic cancer | Height (cm) |
| Prostate cancer | Height (cm) |

**Table S4 – Adjusted associations between the Lifestyle Index, Polygenic Risk Score, and cancer risk**

| **Cancer Type** |  | **Model 1^a^** |  | **Model 2^b^** | | **Model 3^c,d^** |  |
| --- | --- | --- | --- | --- | --- | --- | --- |
|  | **No. of cases/ person-years** | **HR (95%CI)** | ***P*-value** | **HR (95%CI)** | ***P*-value** | **HR (95%CI)** | ***P*-value** |
| Overall cancer | 15,240/1,926,987 |  |  |  |  |  |  |
| Lifestyle index |  | 0.89 (0.87, 0.90) | <0.0001 | 0.89 (0.87, 0.90) | <0.0001 | - | - |
| PRS |  | 1.09 (1.08, 1.11) | <0.0001 | 1.10 (1.08, 1.11) | <0.0001 | - | - |
| Prostate | 4476/981,844 |  |  |  |  |  |  |
| Lifestyle index |  | 1.04 (1.01, 1.08) | 0.0051 | 1.04 (1.01, 1.07) | 0.0082 | 1.04 (1.01, 1.07) | 0.0080 |
| PRS |  | 1.36 (1.32, 1.40) | <0.0001 | 1.36 (1.32, 1.40) | <0.0001 | 1.36 (1.32, 1.40) | <0.0001 |
| Colorectal | 2150/1,990,006 |  |  |  |  |  |  |
| Lifestyle index |  | 0.85 (0.82, 0.89) | <0.0001 | 0.85 (0.82, 0.89) | <0.0001 | 0.85 (0.82, 0.89) | <0.0001 |
| PRS |  | 1.36 (1.31, 1.41) | <0.0001 | 1.36 (1.31, 1.42) | <0.0001 | 1.36 (1.31, 1.42) | <0.0001 |
| Post-menopausal breast | 1990/576,641 |  |  |  |  |  |  |
| Lifestyle index |  | 0.85 (0.81, 0.89) | <0.0001 | 0.85 (0.81, 0.89) | <0.0001 | 0.85 (0.81, 0.89) | <0.0001 |
| PRS |  | 1.26 (1.20, 1.31) | <0.0001 | 1.26 (1.20, 1.31) | <0.0001 | 1.26 (1.20, 1.32) | <0.0001 |
| Lung | 1256/1,994,812 |  |  |  |  |  |  |
| Lifestyle index |  | 0.57 (0.54, 0.61) | <0.0001 | 0.57 (0.54, 0.61) | <0.0001 | 0.59 (0.56, 0.62) | <0.0001 |
| PRS |  | 1.26 (1.19, 1.33) | <0.0001 | 1.26 (1.19, 1.33) | <0.0001 | 1.26 (1.19, 1.33) | <0.0001 |
| Melanoma | 1200/1,994,915 |  |  |  |  |  |  |
| Lifestyle index |  | 1.05 (0.99, 1.12) | 0.076 | 1.05 (0.99, 1.12) | 0.082 | 1.04 (0.98, 1.11) | 0.19 |
| PRS |  | 1.39 (1.32, 1.47) | <0.0001 | 1.39 (1.32, 1.47) | <0.0001 | 1.41 (1.33, 1.49) | <0.0001 |
| Non-Hodgkin lymphoma | 721/1,997,000 |  |  |  |  |  |  |
| Lifestyle index |  | 0.99 (0.92, 1.07) | 0.77 | 0.99 (0.92, 1.07) | 0.75 | 0.99 (0.92, 1.07) | 0.79 |
| PRS |  | 1.19 (1.11, 1.28) | <0.0001 | 1.19 (1.11, 1.28) | <0.0001 | 1.19 (1.11, 1.28) | <0.0001 |
| Kidney | 547/1,997,962 |  |  |  |  |  |  |
| Lifestyle index |  | 0.78 (0.72, 0.85) | <0.0001 | 0.78 (0.72, 0.85) | <0.0001 | 0.78 (0.72, 0.85) | <0.0001 |
| PRS |  | 1.14 (1.05, 1.24) | 0.0019 | 1.14 (1.05, 1.24) | 0.0018 | 1.14 (1.05, 1.24) | 0.0019 |
| Uterine | 494/995,300 |  |  |  |  |  |  |
| Lifestyle index |  | 0.82 (0.74, 0.89) | <0.0001 | 0.82 (0.74, 0.89) | <0.0001 | 0.82 (0.74, 0.90) | <0.0001 |
| PRS |  | 1.31 (1.20, 1.43) | <0.0001 | 1.31 (1.20, 1.43) | <0.0001 | 1.31 (1.20, 1.44) | <0.0001 |
| Pancreatic | 451/1,998,456 |  |  |  |  |  |  |
| Lifestyle index |  | 0.79 (0.72, 0.87) | <0.0001 | 0.79 (0.72, 0.87) | <0.0001 | 0.79 (0.72, 0.87) | <0.0001 |
| PRS |  | 1.51 (1.38, 1.66) | <0.0001 | 1.51 (1.38, 1.66) | <0.0001 | 1.51 (1.38, 1.66) | <0.0001 |
| Bladder | 424/1,998,387 |  |  |  |  |  |  |
| Lifestyle index |  | 0.72 (0.65, 0.79) | <0.0001 | 0.72 (0.65, 0.79) | <0.0001 | - | - |
| PRS |  | 1.21 (1.10, 1.33) | 0.0001 | 1.21 (1.10, 1.33) | 0.0001 | - | - |
| Oral cavity/ pharyngeal | 415/1,998,464 |  |  |  |  |  |  |
| Lifestyle index |  | 0.78 (0.71, 0.86) | <0.0001 | 0.78 (0.71, 0.86) | <0.0001 | 0.79 (0.71, 0.87) | <0.0001 |
| PRS |  | 1.03 (0.93, 1.13) | 0.56 | 1.03 (0.94, 1.13) | 0.54 | 1.03 (0.94, 1.14) | 0.52 |
| Ovarian | 315/996,236 |  |  |  |  |  |  |
| Lifestyle index |  | 0.99(0.88, 1.11) | 0.81 | 0.99 (0.88, 1.11) | 0.82 | 0.99 (0.87, 1.12) | 0.84 |
| PRS |  | 1.08 (0.97, 1.21) | 0.14 | 1.08 (0.97, 1.21) | 0.14 | 1.09 (0.97, 1.21) | 0.16 |
| Lymphocytic leukaemia | 246/1,999,253 |  |  |  |  |  |  |
| Lifestyle index |  | 0.99 (0.87, 1.13) | 0.90 | 1.00 (0.88, 1.13) | 0.96 | - | - |
| PRS |  | 1.50 (1.33, 1.69) | <0.0001 | 1.50 (1.33, 1.69) | <0.0001 | - | - |

| Overall cancer = Overall incident cases of the 13 cancer types assessed in this study; PRS = Polygenic Risk Score  ^a^ Cox proportional hazards regression adjusted for age at baseline, sex (where relevant), assessment centre, 40 principal components of ancestries (PCs), Townsend Index, education, birth location, and income  ^b^ Model 1 + PRS or lifestyle index  ^c^ Model 2 + additional cancer-specific covariates as per Table S3.  ^d^ Note: Cases and person-years in Model 3 differed slightly for some cancers due to missing data for some cancer-specific covariates. |
| --- |

**Table S5 – Relative excess risk due to interaction (RERI) for additive interactions between Lifestyle Index and Polygenic Risk Score for overall cancer and 13 cancer types**

| Lifestyle Index | **Polygenic Risk Score** | | | | | | **Overall test for RERI**  **(*P*-value)** |
| --- | --- | --- | --- | --- | --- | --- | --- |
|  | **Intermediate** | | | **Higher** | | |  |
|  | **RERI** | **95%CI** | ***P*-value** | **RERI** | **95%CI** | ***P*-value** |  |
| **Overall cancer** |  |  |  |  |  |  | 0.049 |
| Intermediate | 0.07 | -0.05, 0.18 | 0.24 | -0.007 | -0.13, 0.11 | 0.91 |  |
| Lower | 0.04 | -0.07, 0.16 | 0.45 | 0.08 | -0.04, 0.20 | 0.17 |  |
| **Prostate cancer** |  |  |  |  |  |  | 0.14 |
| Intermediate | 0.22 | 0.007, 0.43 | 0.043 | 0.16 | -0.08, 0.41 | 0.20 |  |
| Lower | 0.06 | -0.14, 0.26 | 0.57 | -0.09 | -0.33, 0.15 | 0.46 |  |
| **Colorectal cancer** |  |  |  |  |  |  | <0.0001 |
| Intermediate | 0.32 | -0.03, 0.67 | 0.071 | 0.46 | 0.08, 0.85 | 0.017 |  |
| Lower | 0.25 | -0.11, 0.61 | 0.18 | 0.61 | 0.22, 0.99 | 0.0021 |  |
| **Post-menopausal breast cancer** |  |  |  |  |  |  | 0.0001 |
| Intermediate | -0.09 | -0.51, 0.33 | 0.68 | 0.22 | -0.21, 0.65 | 0.32 |  |
| Lower | -0.04 | -0.47, 0.39 | 0.86 | 0.43 | -0.02, 0.88 | 0.059 |  |
| **Lung cancer** |  |  |  |  |  |  | <0.0001 |
| Intermediate | 0.43 | -0.08, 0.94 | 0.098 | 0.53 | -0.009, 1.08 | 0.054 |  |
| Lower | 1.21 | 0.60, 1.82 | 0.0001 | 1.96 | 1.25, 2.67 | <0.0001 |  |
| **Melanoma** |  |  |  |  |  |  | 0.40 |
| Intermediate | 0.46 | 0.07, 0.84 | 0.021 | 0.08 | -0.39, 0.55 | 0.74 |  |
| Lower | 0.43 | 0.08, 0.78 | 0.016 | 0.25 | -0.19, 0.68 | 0.26 |  |
| **Non-Hodgkin lymphoma** |  |  |  |  |  |  | 0.67 |
| Intermediate | 0.59 | 0.21, 0.97 | 0.0023 | 0.20 | -0.26, 0.66 | 0.40 |  |
| Lower | 0.51 | 0.13, 0.89 | 0.0090 | -0.08 | -0.56, 0.39 | 0.74 |  |
| **Kidney cancer** |  |  |  |  |  |  | 0.0049 |
| Intermediate | -0.37 | -1.47, 0.72 | 0.50 | -0.34 | -1.46, 0.78 | 0.55 |  |
| Lower | -0.12 | -1.11, 0.87 | 0.81 | 0.76 | -0.22, 1.73 | 0.13 |  |
| **Uterine cancer** |  |  |  |  |  |  | 0.098 |
| Intermediate | 0.69 | -0.06, 1.44 | 0.073 | 0.14 | -0.74, 1.03 | 0.75 |  |
| Lower | 0.92 | 0.06, 1.79 | 0.037 | -0.04 | -1.02, 0.95 | 0.94 |  |
| **Pancreatic cancer** |  |  |  |  |  |  | 0.0002 |
| Intermediate | 0.38 | -0.68, 1.44 | 0.48 | 0.48 | -0.68, 1.65 | 0.42 |  |
| Lower | 0.70 | -0.34, 1.74 | 0.19 | 1.55 | 0.33, 2.77 | 0.013 |  |
| **Bladder cancer** |  |  |  |  |  |  | 0.0002 |
| Intermediate | 0.07 | -0.81, 0.96 | 0.87 | 0.31 | -0.55, 1.17 | 0.48 |  |
| Lower | 0.73 | -0.14, 1.59 | 0.099 | 1.12 | 0.25, 1.99 | 0.012 |  |
| **Oral cavity/pharyngeal cancer** |  |  |  |  |  |  | 0.73 |
| Intermediate | 0.57 | -0.03, 1.17 | 0.061 | 0.13 | -0.53, 0.79 | 0.69 |  |
| Lower | 0.44 | -0.18, 1.06 | 0.17 | 0.10 | -0.58, 0.78 | 0.77 |  |
| **Ovarian cancer** |  |  |  |  |  |  | 0.27 |
| Intermediate | 0.08 | -0.62, 0.78 | 0.81 | 0.65 | -0.03, 1.33 | 0.060 |  |
| Lower | 0.15 | -0.56, 0.85 | 0.69 | 0.44 | -0.24, 1.12 | 0.21 |  |
| **Lymphocytic leukaemia** |  |  |  |  |  |  | 0.69 |
| Intermediate | -0.36 | -1.42, 0.69 | 0.50 | 0.95 | -0.14, 2.04 | 0.086 |  |
| Lower | -0.40 | -1.43, 0.64 | 0.45 | 0.49 | -0.53, 1.51 | 0.35 |  |

Overall cancer = overall incident cases of the 13 cancer types assessed in this study

Cox proportional hazards regression models adjusted for age at baseline, sex (where relevant), assessment centre, 40 principal components of ancestries, Townsend Index, education, birth location, and income

**References**

1. Alcohol Change UK. Alcohol units. Accessed February 23, 2021. <https://alcoholchange.org.uk/alcohol-facts/interactive-tools/check-your-drinking/alcohol-units>.

2. The IPAQ Group. Guidelines for the data processing and analysis of the International Physical Activity Questionnaire (IPAQ) – short and long forms. Accessed February 25, 2021. <https://www.researchgate.net/file.PostFileLoader.html?id=5641f4c36143250eac8b45b7&assetKey=AS%3A294237418606593%401447163075131>.
